# Emergence and transmission of Carbapenem-resistant *Enterobacteriaceae bla*_NDM-5_ gene in healthy pigs in Baise, Guangxi, China: a discovery

**DOI:** 10.3389/fvets.2025.1501997

**Published:** 2025-05-07

**Authors:** Yuan-Yuan Dai, Wen-Fei Wei, Xue-zhen Li, Chao-Yuan Yuan, Li-Juan Zhao, Yan-Qiang Huang, Ming Hao, Ying Deng, Yuan-Ji Teng, Xue-Li Yi

**Affiliations:** ^1^Affiliated Hospital of Youjiang Medical University for Nationalities, Baise, China; ^2^Center for Medical Laboratory Science, Affiliated Hospital of Youjiang Medical University for Nationalities, Guangxi, China; ^3^Baise Key Laboratory for Research and Development on Clinical Molecular Diagnosis for High-Incidence Diseases, Guangxi, China; ^4^Key Laboratory of Research on Clinical Molecular Diagnosis for High Incidence Diseases in Western Guangxi of Guangxi Higher Education Institutions, Guangxi, China; ^5^College of Animal Science and Technology, Guangxi University, Nanning, China; ^6^Baise Center for Animal Disease Prevention and Control, Baise, China; ^7^Baise Agriculture and Rural Bureau, Baise, China; ^8^School of Basic Medicine, Youjiang Medical University for Nationalities, Baise, China; ^9^Guangxi Zhuang Autonomous Region Engineering Research Center of Clinical Prevention and Control Technology and Leading Drug for Microorganisms with Drug Resistance in Border Ethnic Areas, Baise, China; ^10^Key Laboratory of the Prevention and Treatment of Drug-Resistant Microbial Infecting, Youjiang Medical University for Nationalities, Education Department of Guangxi Zhuang Autonomous Region, Baise, China

**Keywords:** CRE, carbapenems, *bla*_NDM_, resistance transmission, swine

## Abstract

A total of 366 tonsillar tissue samples were collected from healthy free-range pigs owned by farmers across 12 districts in Baise City, Guangxi, China. This initiative successfully isolated six strains of Carbapenem-Resistant *Enterobacteriaceae* (CRE), including four strains of *Escherichia coli*, and one of *Klebsiella aerogenes* and *Morganella morganii*. Assessments utilizing Carbapenemase inhibitor enhancement test, in conjunction with the ResFinder resistance gene database, revealed that five strains carried the *bla*_NDM-5_ gene, while one strain possessed the *bla*_NDM-1_ gene. The overall positivity rate for CRE was determined to be 1.64%. Conjugation tests further demonstrated the ability of the *bla*_NDM_ genes to transfer to recipient strains. Additionally, an analysis of the genetic environment surrounding the *bla*_NDM_ genes showed similarities among the genes within the same geographic region, suggesting that these strains may have originated from a common ancestor and spread horizontally among different clones. This study provides valuable insights into the transmission of CRE among free-range farmers in Baise City, Guangxi, China.

## 1 Introduction

“One Health” refers to the overall health of humans, animals, and the environment, to achieve harmonious unity of human health, animal health, and environmental health. Antibiotics, as one of the major inventions of the 20th century, have played a significant role in medical, livestock, and agricultural fields. However, livestock farms are the epicenter of antibiotic use, with nearly two-thirds of antibiotics being used in livestock production (1). It has been reported that the global antibiotic consumption rate was approximately 15% in 2018, and the daily antibiotic usage per 1,000 people increased by 46% compared to the year 2000 (2). This overuse of antibiotics has led to the rapid growth of Anti-Microbial Resistance (AMR), which reduces the effectiveness of antimicrobial drugs, and presents unprecedented challenges for human anti-infective therapy (3). Studies have found that by 2050, 10 million people will die from AMR infections (4). Therefore, AMR has been identified by the World Health Organization (WHO) as one of the public health threats to human health, and there is a call for action to curb its spread (5). The “One Health” concept is a key strategy to address this multi-sectoral public health crisis (6, 7).

Under the selective pressure of antibiotics, bacteria continuously evolve new mechanisms of resistance, leading to the emergence of new antibiotic resistance genes (ARGs). Bacteria carrying ARGs are considered a “gene reservoir” and can rapidly spread through the food chain and ecosystems, affecting the health of humans, animals, and the environment (8). In livestock farming, antibiotics are primarily used to promote animal growth and prevent and treat diseases. Due to the incomplete absorption of antibiotics by animals, the environment through excretion in their original or metabolized forms, thereby promoting the spread of antibiotic-resistant bacteria and ARGs. ARGs are typically present on bacterial plasmids and continuously self-replicate, and then spread to the surrounding environment through horizontal transfer, making the surrounding environment a key link in the generation and spread of antibiotic-resistant bacteria.

Carbapenems are a class of broad-spectrum *β*-lactam antibiotics with extremely potent antibacterial activity against bacteria of the *Enterobacteriaceae* family. They are also considered the last line of defense in clinical treatment against multidrug-resistant Gram-negative bacteria (9). With the use of carbapenem antibiotics, Carbapenem-Resistant *Enterobacterales* (CRE) have been successively discovered (10). In 2014, the WHO found that the resistance rates of carbapenem-resistant *Klebsiella pneumoniae* (CRKP) in Europe, Southeast Asia, and other regions had reached as high as 70 and 55% (11). In 2013, the mortality rate of CRE in North Carolina, USA, reached 50% (12). However, the prevalence of CRE in China has reached 15%, with a related mortality rate of 33.5% (13). Carbapenem-resistant strains are highly resistant, have a high mortality rate, and spread rapidly, contradicting “the One Health” concept. Therefore, in 2017, the WHO listed CRE as the top resistant pathogen posing the greatest threat to human health and urgently requiring new antimicrobial drugs to combat its spread (14).

Although the use of carbapenem antibiotics in animal husbandry has not been approved, this has not prevented the emergence and spread of CRE in the animal farming environment (15–17). CRE can be transmitted to humans through direct contact, undercooked pork, pig manure, and other pathways. Li et al. (18) first discovered that CRE can be transmitted between humans and animals. Animal farming areas have become important reservoirs for CRE. Various carbapenemase-resistant genes have been found globally, such as *bla*_KPC_, *bla*_NDM_, *bla*_IMP_, and *bla*_VIM_. Since the discovery of New Delhi metallo-*β*-lactamase-1 (NDM-1) in *Klebsiella pneumoniae* by Yong et al. in 2009 (19), bacteria carrying *bla*_NDM_ have been successively discovered worldwide (17, 20–24). *bla*_NDM-5_ was first discovered in *Escherichia coli* (25) and has become one of the widely prevalent variants of *bla*_NDM_ (21, 25, 26). The *bla*_NDM-5_ resistance gene is usually located on mobile genetic elements such as plasmids and transposons, allowing for rapid spread through horizontal gene transfer within bacterial populations (26).

Currently, research on pig-derived CRE in China mainly focuses on pig farms, and relatively few studies have been conducted on the carriage of CRE by farmers’ free-range pigs, and this is especially true for reports on the carriage of CRE by rural free-range pigs in Baise City, Guangxi, China. Therefore, this study aims to screen tonsil samples from free-range pigs raised by rural households in Baise City, Guangxi, to determine the presence of CRE Through conjugation experiments, the study seeks to identify the transmission pathways of relevant resistance genes. This will provide strong experimental evidence for monitoring and studying the transmission mechanisms of CRE in the Baise area, and will aid relevant authorities in formulating effective management strategies to protect public health and safety.

## 2 Materials and methods

### 2.1 Materials

Three hundred and sixty-six samples of pig tonsil tissue, *E. coli* EC600 (Presented by Prof. Binghuai Lu of China-Japan Friendship Hospital), agar powder, Mueller-Hinton agar (M-H) (OXOID, United Kingdom), ultrapure water (UP water) (Solarbio Beijing), Phosphate-Buffered Saline (PBS) (BMC, China), nutrient broth (Solarbio, Beijing), nutrient agar (Solarbio, Beijing), disposable inoculation loops, Eppendorf (EP) tubes, agarose gel electrophoresis apparatus (Bio-Rad, USA), Rifampicin E-test strips (Liofilchem, Italy), Meropenem susceptibility test discs (OXOID, United Kingdom), Rifampicin susceptibility test discs(OXOID, United Kingdom), crystal violet (Solarbio, Beijing), methanol, absolute ethanol, 96-well cell culture plate, *bla*_NDM_ primers, VITEK MS (BioMerieux, France), DNA extraction kit (TaKaRa, China), metal bath, pipettes, VITEK 2 Compact system (BioMerieux, France), thermal cycler, electrophoresis tank, autoclave, shaker, microplate reader, 37°C incubator, gel imaging system, etc.

### 2.2 Experimental methods

#### 2.2.1 Processing of pig samples

Throughout 2023, aseptically collect tonsil tissue samples from healthy free-range pigs raised by rural households in 12 counties (cities, districts) of Baise City, Guangxi, China, totaling 366 samples. The collected tissue blocks were placed in sterile nutrient broth centrifuge tubes, ground, and then stored and sent for inspection under refrigeration.

#### 2.2.2 Isolation and identification of CRE strains

A small amount of ground tissue was inoculated onto China blue agar (Autobio, China) containing 2 μg/mL Meropenem (MCE, USA) and incubated for 24–48 h. Suspected colonies were purified multiple times, identified using VITEK MS and finally, bacteria of the *Enterobacteriaceae* were selected for subsequent experiments.

#### 2.2.3 Minimum inhibitory concentration (MIC) testing of isolated strains

Following the recommendations of the Clinical and Laboratory Standards Institute (CLSI), the MIC of the isolated strains was determined using the broth microdilution method. The VITEK 2 Compact system was used to test 17 antibiotics, and the interpretation of the susceptibility results follows the guidelines provided in the CLSI M100 document, except for tigecycline and polymyxin, which is interpreted according to the European Committee on Antimicrobial Susceptibility Testing (EUCAST) document. CRE refers to *Enterobacteriaceae* bacteria that are resistant to any of the carbapenem antibiotics, including Imipenem, Meropenem, Doripenem, or ertapenem (such as Imipenem, Meropenem, and Doripenem with a MIC≥ 4 μg/mL, or Ertapenem with MIC≥ 2 μg/mL), or those that are confirmed to produce carbapenemases.

#### 2.2.4 Carbapenemase inhibitor enhancement test of isolated strains

The detailed experimental procedure was referred to Hua Yu et al. With minor adjustments (27). Fresh test strains were adjusted to an OD (Optical Density) value of 0.5–0.63 McFarland and evenly spread on the surface of M-H agar. Subsequently, Meropenem discs (10 μg) were placed on the agar surface, containing no additional liquid, one disc with 5 μL of EDTA solution, and another disc with 5 μL of 3-aminophenylboronic acid hydrochloride (APB) solution. The final disc was treated with both EDTA and APB solutions with final concentrations of 300 μg/disc and 292 μg/disc, respectively. After overnight incubation, the size of the inhibition zone was observed.

#### 2.2.5 Bioinformatics analysis

Isolates were subjected to Illumina sequencing (performed by Beijing Novogene Bioinformatics Technology Co., Ltd.) (Bioproject accession ID: PRJNA1229800.). The average nucleotide identity (ANI) values were determined using FastANI (v1.33). Using the databases on the Center for Genomic Epidemiology server,^1^^,^^2^ resistance genes and sequence types (ST) of the isolated strains were predicted to identify potential resistance genes. The VFDB database predicted the virulence factors.^3^ Additionally, the 16S rRNA gene sequence analysis method is used for homology analysis with different species of *Enterobacteriaceae* obtained from NCBI, and the MEGA 7.0 software was used to construct a phylogenetic tree. Finally, the Easyfig 2.2.5 software was used to draw a comparative diagram of the *bla*_NDM_ gene and its surrounding environment.

### 2.3 Conjugation transfer experiment

#### 2.3.1 Preparation of dual antibiotic culture medium and strain recovery

Prepare M-H agar culture medium containing both Meropenem and Rifampicin (final concentrations of 2 μg/mL and 600 μg/mL, respectively) and stored at 4°C. Recover *E. coli* EC600 and the isolated strains by inoculating them into nutrient LB broth (Solarbio, Beijing) and incubating until the logarithmic phase was reached.

#### 2.3.2 Selection of transconjugants

This experiment used specific bacteria as donors and *E. coli* EC600 as acceptor. Following the method of Tang, Kong, et al. (15, 28) with minor adjustments, 0.9 mL of *E. coli* EC600 and 0.3 mL of donor bacteria from step 1.3.1 were mixed in a sterile test tube and then placed in a 37°C incubator for 30 min. After shaking and centrifugation at 4000 rpm, the bacterial liquid is removed, the remaining bacteria are mixed with sterile saline, and finally, 100 μL of the bacterial mixture is placed on a double antibiotic M-H agar medium. Only bacteria grown on a dual antibiotic medium, identified as *E. coli* by mass spectrometry and positive for the *bla*_NDM_ gene by PCR amplification, were considered conjugates. Both the recipient and donor bacteria were used as blank controls. The conjugated strains were named after JNP or JLE (The conjugates of CRE strains isolated from Napo Country or Leye Country with *E. coil* EC600 were named JNP or JLE).

### 2.4 Verification of transconjugants

#### 2.4.1 DNA extraction

Inoculate a single colony grown on the double antibiotic culture medium from step 1.3.2 onto M-H agar and incubate overnight. Transfer an appropriate amount of the bacterial colony to a sterile EP tube containing TE buffer, boil at 100°C for 15 min, then cool at 4°C, and finally centrifuge. Transfer the supernatant to a new sterile EP tube and store it at 4°C, *E. coli* EC600 is used as a blank control.

#### 2.4.2 PCR amplification of target genes

Preparing the reaction system to achieve a total volume of 50 μL for the PCR amplification of the target genes in the transconjugants. Reaction system composed of 2 μL each of forward primer (F) and reverse primer (R), 5 μL of DNA template, 18 μL of double-distilled water (dd H_2_O), and 25 μL of Premix Taq. The primer sequence is CAGCACACTTCCTATCTC (Forward primer) and CCGCAACCATCCCCTCTT (Reverse primer).

#### 2.4.3 Detection of the *bla*_NDM_ gene in transconjugants

Agarose gel electrophoresis is a method used to detect the presence of the *bla*_NDM_ gene in transconjugants. To prepare a 1.5% agarose gel, start by mixing agarose with TE TBE buffer, then heat the mixture until completely dissolved. Let it cool to 50–60°C, add a nucleic acid dye, pour the mixture into the gel casting tray, insert the comb, fill the electrophoresis tank with buffer, and finally, add 5 μL of the DNA amplification product to the gel wells.

#### 2.4.4 MIC of transconjugants

The experimental procedure is the same as in section 1.2.3. The MIC of Rifampicin was determined using E-test strips.

### 2.5 Biofilm formation ability of isolates

Inoculate a sterile centrifuge tube with 8 mL nutrient LB broth using a small number of single colonies and incubate at 37°C until the logarithmic phase. Prepare a 1 × 10^6 CFU/mL bacterial suspension using nutrient LB broth and transfer 200 μL to a 96-well cell plate, inoculating 6 wells for each strain and 6 wells with nutrient LB broth as a negative control. Incubate for 48 h. The steps for biofilm crystal violet staining are as follows: Adjust the bacterial suspension to an OD value between 0.5 and 0.63 McFarland. Then, transfer 200 μL of the bacterial suspension to a cell culture plate and incubate for 36 h at 37°C. After incubation, remove the supernatant from the plate, wash with sterile PBS, and fix the bacterial cells with methanol. Perform another round of washing with PBS and stain the cells with crystal violet for 20 min. Wash the cells with PBS and allow them to air-dry. Add 200 μL of absolute ethanol to the plate, agitate on a shaker, and measure the OD value of the isolates at a wavelength of 580 nm. Conduct each test in 6 replicates and repeat these steps twice, using PBS as the blank control.

### 2.6 Statistical analysis

The data results were processed and analyzed using the “SPSS 26.0” statistical software. Quantitative data was represented using the mean ± standard deviation, and means between two samples were compared using the *t*-test. Comparison among multiple samples was conducted using one-way analysis of variance, with “*p* ≤ 0.05” indicating statistical significance.

## 3 Experimental results

### 3.1 Isolation and identification of CRE

Three hundred and sixty-six samples of ground tonsil tissue from pigs were collected from 12 counties and cultured on China blue agar containing Meropenem. A total of 6 CRE strains were screened, with the majority of CRE strains concentrated in, Leye County and Napo County. The isolated strains were designated as NP6, NP7, NP17, LE2, LE26, and LE29. VITEK MS mass spectrometry identified LE26 and NP7 as *Klebsiella aerogenes* and *Morganella morganii*, while the other 4 strains were identified as *E. coli*. The isolation rate of CRE was 1.6% (6/366), and specific information about the source of positive strains can be found in Table 1.

**Table 1:** Regional distribution of positive samples and CRE isolation situation.

Region (Guangxi)	Origin	Number of samples collected	Isolate of strains	The CRE separation number
Leye County	Healthy pig tonsil	32	3 (9.4%)	LE2 LE6 LE29
Napo County		30	3 (10%)	NP6 NP7 NP17
Tianlin County		32	0	–
Tianyan County		30	0	–
Youjiang region		28	0	–
Tiandong County		33	0	–
Debao County		30	0	–
Pingguo County		30	0	–
Xilin County		30	0	–
Jingxi County		31	0	–
Longlin County		30	0	–
Linyun County		30	0	–

**“CRE” means Carbapenem-Resistant Enterobacteriaceae; “NP” means strains from Napo County; “LE” means strains from Leye County; “–” stands for no CRE strains have been detected in the region.**

### 3.2 MIC of isolates

Table 2 shows the MIC values of the isolates for 18 antibiotics, which were classified into 6 categories. The results showed that the isolates of NP were resistant to Cephalosporins, Quinolones, Tetracyclines, Sulfonamides, and carbapenems, making them multidrug-resistant strains. While the isolates of LE were resistant to Cephalosporins, Sulfonamides, and carbapenems, also classified as multidrug-resistant strains. In addition, the isolates of LE were sensitive to aminoglycosides. All isolates from both regions were sensitive to Amikacin, and except for LE2 and LE26 which were sensitive to Quinolones, the other isolates were resistant (66.7%), except for NP6 which was resistant to Aztreonam (16.7%). Furthermore, all isolates were resistant to Trimethoprim/Sulfamethoxazole, and the *E. coli* EC600 receptor strain was sensitive to all 17 antibiotics.

**Table 2:** The resistance of isolates strains to 18 antibiotics (μg/mL).

Antibiotics	NP6	NP7	NP17	LE2	LE26	LE29	EC600
Ticarcillin/Clavulanate	≥128	≥128	≥128	≥128	≥128	≥128	≤8
Piperacillin/Tazobactam	≥128	16	≥128	≥128	≥128	≥128	≤4
Ceftazidime	≥64	≥64	≥64	≥64	≥64	≥64	0.5
Cefoperazone/Sulbactam	≥64	≥64	≥64	≥64	≥64	≥64	≤8
Cefepime	≥32	≥32	8	8	≥32	8	≤0.12
Aztreonam	16	≤1	≤1	≤1	≤1	≤1	≤1
Imipenem	≥16	≥16	≥16	≥16	≥16	≥16	≤0.25
Meropenem	≥16	≥16	≥16	≥16	≥16	≥16	≤0.25
Amikacin	≤2	≤2	≤2	≤2	≤2	≤2	≤2
Tobramycin	8	≥16	≤1	≤1	≤1	≤1	≤1
Ciprofloxacin	≥4	≥4	≥4	≤0.25	≤0.25	≥4	≤0.25
Levofloxacin	≥8	1	≥8	≤0.12	≤0.12	≥8	0.5
Doxycycline	≥16	≥16	≥16	≥16	4	1	1
Minocycline	4	≥16	≥16	4	8	≤1	≤1
Tigecycline	≤0.5	2	≤0.5	≤0.5	2	≤0.5	≤0.5
Colistin	≤0.5	≥16	≤0.5	≤0.5	≤0.5	≤0.5	≤0.5
Trimethoprim/sulfamethoxazole	≥320	≥320	≥320	≥320	≥320	≥320	≤20
Rifampicin^*^	16	64	4	16	8	8	≥256

^*^E-test strips were used for MIC of Rifampicin.

### 3.3 Carbapenemase inhibitor enhancement assay for isolates

**Figure 1:**
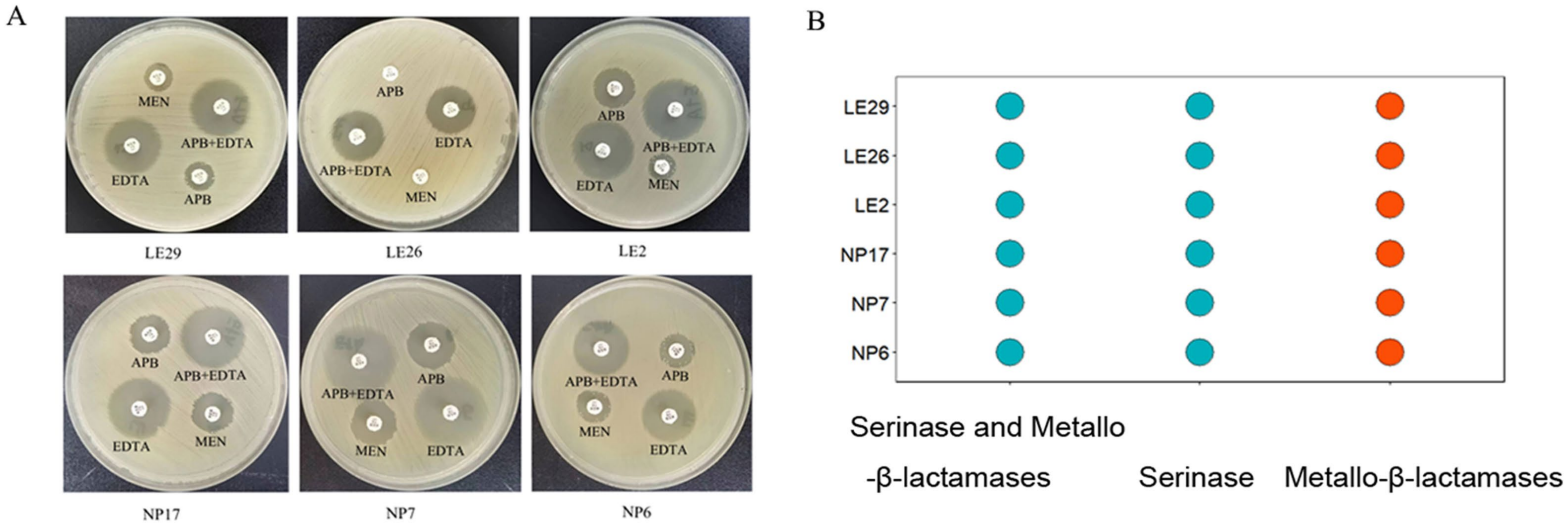
Carbapenemase inhibitor enhancement assay for isolates; **(A)** The difference in the diameter of the circle of inhibition between meropenem paper sheets containing EDTA/APB solution and meropenem paper sheets without EDTA/APB solution was ≥5 mm after 24 h of incubation, indicating that the isolate produced metallo-β-lactamase. It was obvious that all the isolated strains in this experiment produce metallo-β-lactamases; **(B)** #FC4E07 (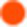) represents the isolated strain produces metallo-β-lactamases.

It is interesting to note that according to the APB-EDTA experiment, all isolated strains produced metallo-*β*-lactamases, and none of them produced serinase or both serinase and metallo-β-lactamases at the same time. This information is illustrated in Figure 1.

### 3.4 Bioinformatics results

#### 3.4.1 MLST, resistant genes, virulence genes prediction results

**Figure 2:**
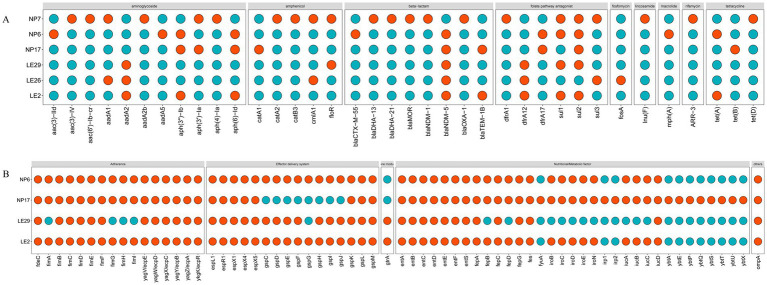
The predict results of the drug-resistant genes and virulence genes. In the figure, **(A)** represents drug-resistant genes, and **(B)** represents virulence. When a gene is present, it was represented by #FC4E07 (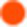); Otherwise, it was represented by #00AFBB (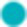). The database cannot provide virulence genes for *Morganella morganii* and *Klebsiella aerogenes.*

It looks like the MLST types of the 4 isolated strains of *Escherichia coli* were ST410, ST2599, and ST3076, while the MLST type of the *Klebsiella aerogenes* was ST349. The ANI results of these strains and the results of MLST typing are shown in Table 3. Figure 2 showed the resistant genes and virulence genes of the isolated strains. Interestingly, all strains carried at least one or more resistant genes and virulence genes, with *bla*_NDM_ being present in all isolated strains, 5 out of 6 isolates carry blaNDM-5 (83.3%) and 1 carries blaNDM-1 (16.7%). The *sul2* resistant gene (83.3%) was found in all *E. coli.* When it comes to adhesion factors, the strain LE29 of *E. coli* lacks certain adhesion factors, while strain NP17 harbors a majority of nutritional/metabolic factors. The effector delivery system was prevalent in *E. coli.*

**Table 3:** MLST types of isolated strains.

Isolation strain number	Strain	ANI value (%)	MLST type
NP6	*Escherichia coli*	96.69	ST2599
NP7	*Morganella morganii*	96.59	–^*^
NP17	*Escherichia coli*	96.91	ST410
LE2	*Escherichia coli*	96.74	ST3076
LE26	*Klebsiella aerogenes*	97.94	ST349
LE29	*Escherichia coli*	96.91	ST410

^*^The database cannot provide the sequence type of *Morganella morganii* MLST; “NP” means strains from Napo County; “LE” means strains from Napo County.

#### 3.4.2 Constructing a phylogenetic tree based on 16S rRNA

**Figure 3:**
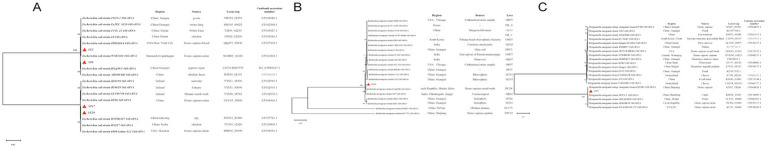
Phylogenetic tree: **(A–C)** are the phylogenetic trees of *Escherichia coli*, *Morganella morganii*, and *Klebsiella aerogenes*, respectively; 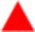 represents the isolated strains.

It seems that in the phylogenetic tree analysis of the isolated strains, shown in Figure 3, there are multiple branches. For *Escherichia coli* (*E. coli*), the ML tree indicates mainly 4 branches. LE2 is closely related to FG31-1 and A320 with a bootstrap value of 99, indicating high homology. NP6 is grouped with fECg99.1 and AHM9C68I, showing high homology with a bootstrap value of 99. All *E. coli* on this branch are from China and isolated from animals, with fECg99.1 originating from Guangxi. NP17, LE29, HNTH2207, HN257, and Ec2 are on the same branch with a bootstrap value of 99, indicating high homology. For *Morganella morganii*, the ML tree shows two main branches. The main branch where NP7 is located is divided into two sub-branches. NP7 is closely related to ZJD581, with a bootstrap value of 100, indicating high homology. For *Klebsiella aerogenes*, there are multiple branches in the ML tree. LE26 is grouped with EA46506 and CH7, showing high homology with a bootstrap value of 100. EA46506 was isolated from human rectal swabs, and CH7 is from earthworm feces, both related to fecal samples.

### 3.5 Isolation strains *bla*_NDM_ surrounding environment

**Figure 4:**
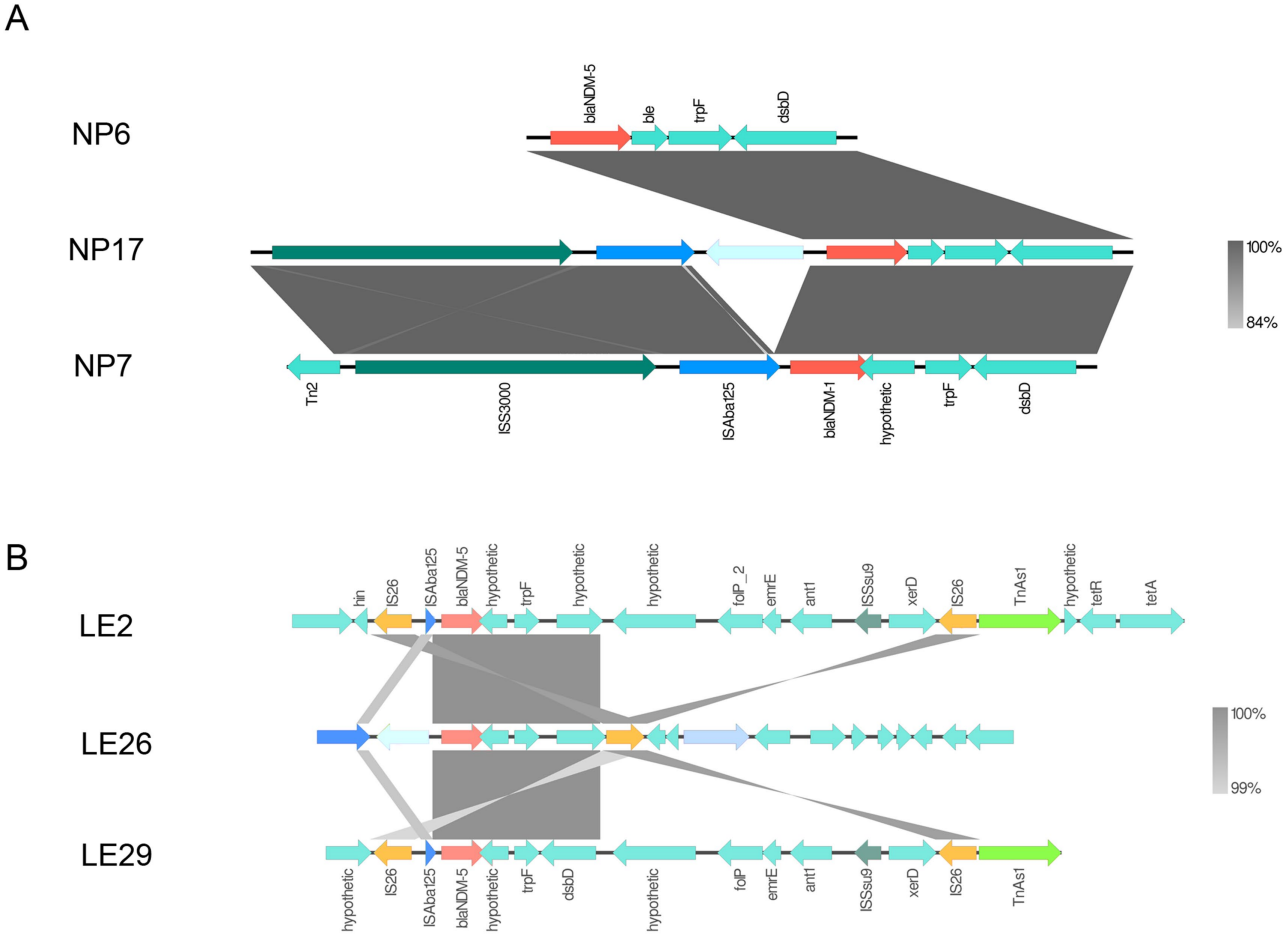
*bla*_NDM_ surrounding environment. Panel **(A)** is the genetic environment diagram of *bla*_NDM_ in Napo County, and **(B)** is the genetic environment diagram of *bla*_NDM_ in Leye County. Regions with over 99% homology are marked in gray.

It’s interesting to note that the contig that contains the *bla*_NDM_ gene was subjected to comparative analysis using the Easyfig software. The analysis showed that isolates from the same region have similar genetic environments surrounding the *bla*_NDM_ gene. In Figure 4A, the genetic environment consists of *Tn2-ISS3000-ISAba125-bla*_NDM_*-trpF-dsbD*, while in Figure 4B, the genetic environment was *IS26-ISAba125-bla*_NDM_*-trpF-dsbD-ISSsu9-IS26-TnAs1*. It’s worth noting that both genetic environments surrounding the *bla*_NDM_ gene contain mobile elements upstream and downstream. Additionally, downstream of LE2, there is also a tetracycline-resistant gene (*tet*A/*tet*R).

### 3.6 MIC of conjugation

It was found that most of the conjugates acquired the resistant phenotype of most of the donor bacteria. All conjugates showed reduced susceptibility to Ticarcillin/Clavulanic acid (6/6), Piperacillin/Tazobactam (6/6), Ceftazidime (6/6), Cefoperazone/Sulbactam (6/6), Cefepime (6/6), Imipenem (6/6), Meropenem (6/6), followed by Ciprofloxacin (2/6), Levofloxacin (2/6), Doxycycline (2/6), Minocycline (2/6), Methotrexate/Sulfamethoxazole (1/6), Amitraz (1/6), Tobramycin (1/6). There was no significant change in the sensitivity of Amikacin, Tigecycline and Colistin after conjugation (as shown in Table 4).

**Table 4:** The resistance of conjugations to 18 antibiotics (μg/mL).

Antibiotics	EC600	NP6	JNP6	NP7	JNP7	NP17	JNP17	LE2	JLE2	LE26	JLE26	LE29	JLE29
Ticarcillin/Clavulanate	≤8	≥128	≥128	≥128	≥128	≥128	≥128	≥128	≥128	≥128	≥128	≥128	≥128
Piperacillin/Tazobactam	≤4	≥128	≥128	16	≥128	≥128	≥128	≥128	≥128	≥128	≥128	≥128	≥128
Ceftazidime	0.5	≥64	≥64	≥64	≥64	≥64	≥64	≥64	≥64	≥64	≥64	≥64	≥64
Cefoperazone/Sulbactam	≤8	≥64	≥64	≥64	≥64	≥64	≥64	≥64	≥64	≥64	≥64	≥64	≥64
Cefepime	≤0.12	≥32	4	≥32	2	8	16	8	2	≥32	–	8	8
Aztreonam	≤1	16	16	≤1	≤1	≤1	≤1	≤1	≤1	≤1	≤1	≤1	≤1
Imipenem	≤0.25	≥16	8	≥16	8	≤0.25	≥16	≥16	≥16	≥16	≥16	≥16	8
Meropenem	≤0.25	≥16	≥16	≥16	≥16	8	≥16	≥16	≥16	≥16	≥16	≥16	≥16
Amikacin	≤2	≤2	≤2	≤2	≤2	≤2	≤2	≤2	≤2	≤2	≤2	≤2	≤2
Tobramycin	≤1	8	2	≥16	≤1	≤1	≤1	≤1	≤1	≤1	≤1	≤1	≤1
Ciprofloxacin	≤0.25	≥4	≥4	≥4	≤0.25	≥4	≤0.25	≤0.25	≤0.25	≤0.25	≤0.25	≥4	≥4
Levofloxacin	0.5	≥8	≥8	1	0.5	≥8	0.5	≤0.12	≤0.12	≤0.12	0.5	≥8	≥8
Doxycycline	1	≥16	≥16	≥16	1	≥16	1	≥16	≥16	4	1	1	2
Minocycline	≤1	4	8	≥16	≤1	≥16	≤1	4	8	8	≤1	≤1	≤1
Tigecycline	≤0.5	≤0.5	≤0.5	2	≤0.5	≤0.5	≤0.5	≤0.5	≤0.5	2	≤0.5	≤0.5	≤0.5
Colistin	≤0.5	≤0.5	≤0.5	≥16	≤0.5	≤0.5	≤0.5	≤0.5	≤0.5	≤0.5	≤0.5	≤0.5	≤0.5
Trimethoprim/sulfamethoxazole	≤20	≥320	≤20	≥320	≤20	≥320	≤20	≥320	≥320	≥320	≤20	≥320	≤20
Rifampicin^*^	≥256	16	≥256	64	≥256	4	≥256	16	≥256	8	≥256	8	≥256

^*^E-test strips were used for MIC of Rifampicin. The conjugates of CRE strains isolated from Napo Country or Leye Country with *E. coli* EC600 named JNP or JLE.

### 3.7 Detection of conjugative *bla*_NDM_ gene

**Figure 5:**
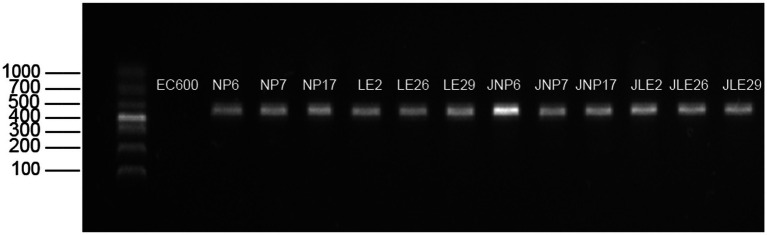
Agarose gel electrophoresis detection of *bla*_NDM_ resistance gene.

It seems like the experiment was successful in transferring the *bla*_NDM_ gene to the recipient cells via conjugation. The conjugative cells were able to grow on the double-antibiotic MH agar medium, and the amplification product of the conjugative cells was consistent with the size of the target gene. Gel electrophoresis confirmed that all cells after conjugation carried the *bla*_NDM_ resistance gene, which is also consistent with the predictions made by the ResFinder database. The results of the conjugative *bla*_NDM_ gene are depicted in Figure 5.

### 3.8 Biofilm formation ability of isolates

**Figure 6:**
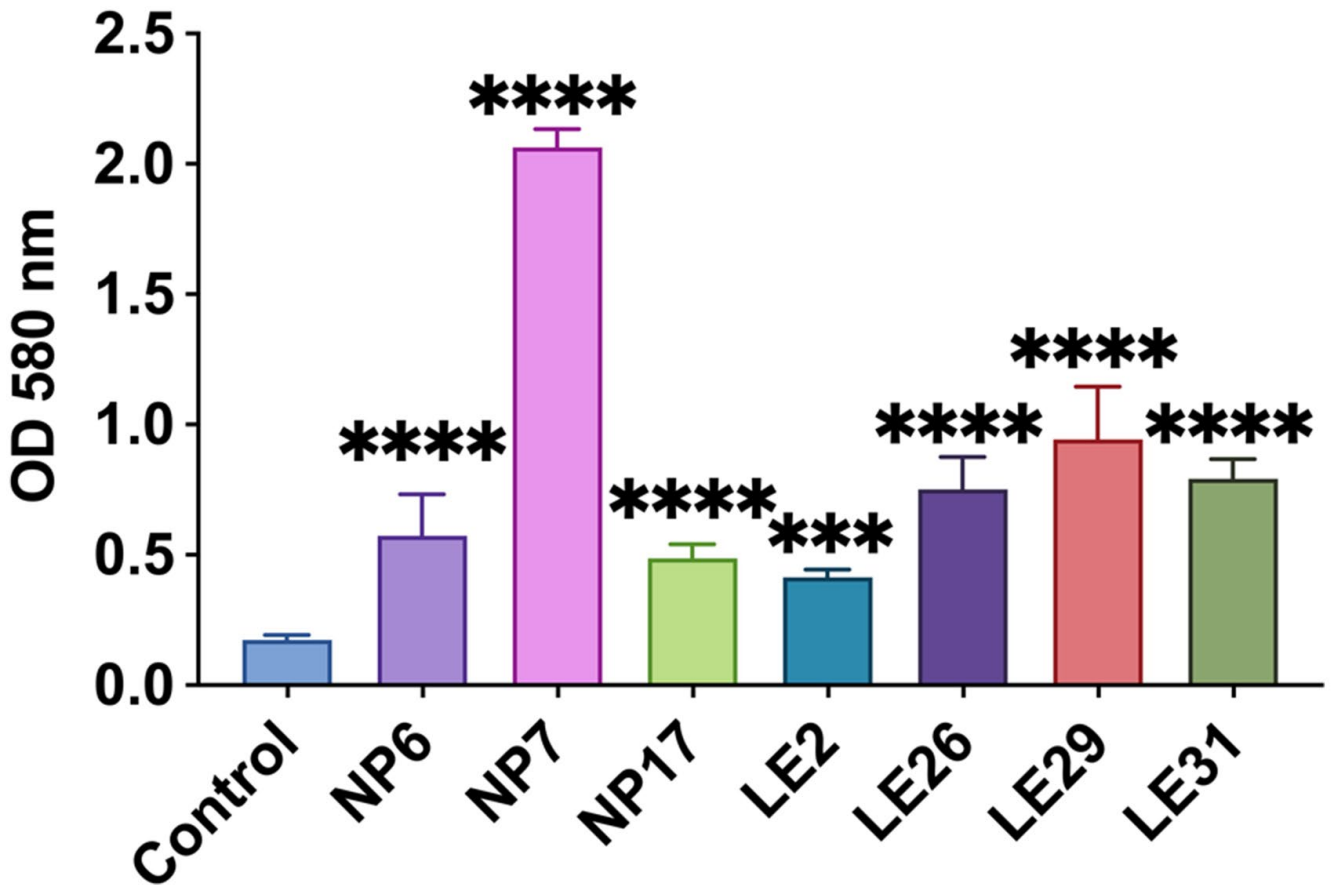
Biofilm formation experiment; “***” stands for *p* ≤ 0.01; “****” stands for *p* ≤ 0.001.

It appears that in this experiment, all the isolates were found to be capable of forming biofilms *in vitro*. Notably, there was a statistically significant difference observed in comparison to the control group (*p* < 0.05), as depicted in Figure 6. These findings suggest that biofilm formation could be an important mechanism of bacterial resistance in this context.

## 4 Discussion and conclusion

The overuse of antibacterial drugs has led to a rise in resistance that poses a significant threat to public health. There have been reports of CRE being isolated from hospitals, livestock, food, and the environment (15, 18, 26, 29, 30). In a recent study conducted in Baise City, China, six strains of CRE were isolated from free-range farmers, with a detection rate of 1.6%. Four of the isolates were identified as *E. coli*, while the other two were *Morganella morganii* and *Klebsiella aerogenes*. Analysis revealed that all isolates displayed resistance to the majority of *β*-lactam antibiotics, with NP7 demonstrating additional resistance to polymyxins, which are typically considered the last line of defense against CRE infection. The prevalence of polymyxin resistance in domestic animals and pigs has been associated with the rise and dissemination of the mcr-1 gene, often found on plasmids (31, 32). If the administration of polymyxin antibiotics in livestock is not regulated, the likelihood of mcr-1 resistance spreading from animals to humans is high, potentially limiting treatment options for CRE infections.

The results of the carbapenemase inhibition assays indicate that all isolated strains produce Class B enzymes, with *bla*_NDM_ being the most common resistance gene among CRE strains in China. Additionally, resistance gene prediction is consistent with this result, as 5 out of 6 isolates carry *bla*_NDM-5_ (83.3%) and 1 carries *bla*_NDM-1_. It’s worth noting that *bla*_NDM-5_ is a newer variant that demonstrates stronger resistance to carbapenem drugs compared to *bla*_NDM-1_. Moreover, more *bla*_NDM_ variants have been identified since the discovery of new variants of metallo-*β*-lactamases in 2009 (33). It’s crucial to continuously monitor whether *bla*_NDM-5_-carrying strains are predominant among locally healthy pigs, as indicated by experimental results.

In the study, isolated *E. coli* strains can be grouped into three different STs, as ST410, ST2599, and ST3076. MLST type of the CR *Klebsiella aerogenes* was ST349, it was found that two out of the four CRECO isolates were of the ST410 type, accounting for 50% of the total. Various ST types of CRECO are prevalent worldwide, including ST101, ST405, ST410, ST167, and ST131 (34). In China, the most common ST types of CRECO from 2015 to 2017 were ST167, ST131, and ST410, with most of these types carrying the *bla*_NDM_ resistance gene (35). Furthermore, Peng C research has confirmed that the predominant type of CRECO is ST410, with most CRECO strains carrying the *bla*_NDM_ resistance gene (36). The phylogenetic tree also revealed a high homology between the isolated CRE strains in this study and those reported from human and animal sources elsewhere. This suggests that ST410 is widely distributed globally and has the potential to be transmitted to animals through the food chain. Otherwise, we also found that different isolate strains of STs carry multiple resistance and virulence genes.

We used agarose gel electrophoresis to detect the *bla*_NDM_ gene. For large quantities of samples, we can also use recombinase polymerase amplification (RPA), which is currently a fast, easy, and temperature-controlled method (37). Numerous studies have demonstrated that the *bla*_NDM_ gene, which is accountable for antibiotic resistance in bacteria, is predominantly carried on plasmids and can be transferred between different bacterial species (30). This presents a significant risk for the widespread dissemination of antibiotic resistance. Additionally, *bla*_NDM_ is typically situated within transposons rich in *IS26* insertion sequences and *ISCR27* (38). With the increasing understanding of CRE, it has been observed that isolated *bla*_NDM_ strains often carry partial or complete *ISAba125* (39), which provides the promoter region for *bla*_NDM_, and *IS26* plays a significant role in the formation of *bla*_NDM_ multidrug resistance cassettes (40). The mobility of *bla*_NDM_ between bacteria using mobile genetic elements makes it challenging to control the spread of antibiotic-resistant bacteria. To confirm this, conjugation experiments and verification were conducted on the isolated strains. The experimental results, based on minimum inhibitory concentration and agarose gel electrophoresis, indicate that *bla*_NDM_ in donor cells can indeed be transferred to recipient cells through conjugation. This suggests that the *bla*_NDM_ gene can be transmitted between bacterial species through mobile elements, consistent with previous findings (29). Furthermore, the gene environments of the isolated strains consisted of two main types, both of which had mobile elements located upstream of the *bla*_NDM_ mobile element. Analysis of the genetic environment of the isolated *bla*_NDM_ strains revealed that those from the same geographic environment had similar genetic environments. The experimental results confirmed that the gene can be transferred between bacteria and may be associated with mobile elements such as IS26 and ISAba125, whose presence accelerates the transmission of *bla*_NDM_ in different bacteria and environments, posing an uncontrollable risk for CRE transmission.

The role of plasmids in transmitting resistance genes is indeed significant. A study by Yang QE et al. (29) found that the plasmid pX3-NDM-5 has a broad host range. When transferred to *Enterococcus*, it was capable of recombining back into *Escherichia coli*, demonstrating its ability to spread across different bacterial phyla and providing insights into the spread of antibiotic resistance in humans. Other studies have shown that the IncX3 plasmid efficiently carries the *bla*_NDM-5_ gene (41, 42). However, it’s worth noting that our experiment did not conduct whole-genome sequencing on the isolated strains, which is a limitation of our study. Future experiments will undoubtedly enhance our understanding of CRE strain plasmids.

It is worth noting that the strains identified during the study were capable of forming biofilms *in vitro*. These biofilms serve as a protective barrier for bacteria against antibiotics, allowing them to adhere and thrive in diverse environments, including within hosts. The virulence factors of each strain were categorized, as shown in Figure 3. All carry fimA and fimC proteins except LE29, which does not carry fimA protein. Adhesion factors play a role in biofilm formation. Studies have shown that fim proteins are involved in *Klebsiella pneumoniae* biofilm formation (42). Blumer C showed that *LrhA* affects *E. coli* biofilm formation by regulating fim proteins (43, 44). However, the study did not explore the specific molecular mechanisms underlying the formation of these biofilms by CRE strains. In the future, further research could investigate these mechanisms, along with regulatory factors and their connections to antibiotic resistance. This could lead to a deeper comprehension of the pathogenic mechanisms of CRE strains, offering valuable theoretical and practical insights for the prevention and management of CRE-related infections.

Our research underscores the critical need for more effective measures to prevent and control antibiotic resistance in animal-derived CRE. By providing valuable new evidence on the prevalence of antibiotic resistance genes, we can formulate comprehensive strategies to protect public health. However, further research is essential to comprehend the spread of *bla*_NDM_ in animal populations and to devise more effective control strategies. Embracing the “One Health” framework allows us to integrate knowledge and resources from various disciplines to develop comprehensive prevention and intervention strategies that can effectively curb the development of bacterial resistance, thereby reducing the risks to human society and the ecological environment.

In the Guangxi Baise region, healthy pigs were discovered to harbor CRE strains containing transferable *bla*_NDM_ genes on mobile genetic elements. The presence of the *bla*_NDM_ genetic environment in pigs from the same geographic area implies a potential common ancestry and horizontal spread to distinct clones, which is concerning. This research provides insights into the transmission of CRE across livestock farms and underscores the importance of implementing measures to prevent its dissemination to safeguard the well-being of both animals and humans.

## Data Availability

The data in this study have been stored in the NCBI repository, and the accession numbers of these strains are JBLWNH000000000, JBLWNI000000000, JBLWNJ000000000, JBLWNK000000000, JBLWNL000000000 and JBLWNM000000000, respectively.

## Footnotes

^1^ https://cge.food.dtu.dk/services/ResFinder/

^2^ https://cge.food.dtu.dk/services/MLST/

^3^ https://www.mgc.ac.cn/VFs/
